# Integrating Phenotypic and Genotypic Approaches to Select Rust- and Common Bunt-Resistant Advanced Winter Wheat Breeding Lines

**DOI:** 10.3390/plants15081258

**Published:** 2026-04-19

**Authors:** Gaziza Zhumaliyeva, Bakyt Ainebekova, Tamara Bazylova, Assel Jenisbayeva, Ayazhan Kosshybay, Saltanat Dubekova, Raushan Yerzhebayeva

**Affiliations:** 1 Laboratory of Plant Biotechnology, Kazakh Research Institute of Agriculture and Plant Growing, Almaty District, Almalybak 040909, Kazakhstan; zh.gaziza95@gmail.com (G.Z.); t.bazylova@mail.ru (T.B.); jenisbayeva@gmail.com (A.J.); ayazhan.kosshybay@alumni.nu.edu.kz (A.K.); 2 Cereal Crops Laboratory, Kazakh Research Institute of Agriculture and Plant Growing, Almaty District, Almalybak 040909, Kazakhstan; bakyt.alpisbay@gmail.com; 3 Laboratory of Immunity and Plant Protection, Kazakh Research Institute of Agriculture and Plant Growing, Almaty District, Almalybak 040909, Kazakhstan; dubekovasaltanat41@gmail.com

**Keywords:** winter wheat, breeding line, rust, common bunt, molecular marker, resistance gene, infection background

## Abstract

In major wheat-growing regions, rust diseases and common bunt significantly reduce wheat productivity, especially in years with favorable conditions for phytopathogen development and limited resistant cultivar use. Thus, the development of genetically resistant wheat cultivars carrying combinations of valuable resistance genes is an effective strategy to mitigate these losses. In this study, 156 advanced winter wheat breeding lines were evaluated for resistance to yellow (stripe) rust, leaf (brown) rust, and common bunt under an artificial infection background. Concurrently, molecular screening was performed using DNA markers to detect rust (*Yr5*, *Yr10*, *Yr15*, *Lr9*, *Lr34*/*Yr18*, and *Lr37*/*Yr17*) and common bunt resistance genes (*Bt8*, *Bt9*, *Bt10*, *Bt11*, and *Bt12*). Based on the integrated analysis of phenotypic and DNA marker-based molecular data, fourteen and five lines resistant to common bunt and yellow rust, respectively, were identified, and alleles associated with resistance were also detected. Notably, one line (9909) exhibited high resistance to both rust diseases and common bunt. These selected advanced breeding lines represent promising candidates for the development of wheat cultivars with enhanced disease resistance, thereby supporting sustainable productivity in wheat-growing regions.

## 1. Introduction

Wheat (*Triticum aestivum* L.) is one of the most important cereal crops and a staple food in many regions of the world [1,2]. Due to its wide distribution, high adaptability, and versatility, wheat plays a crucial role in ensuring global food security and the sustainability of agroecosystems [3].

Wheat is considered a vital food in Central Asia [4,5], where the per capita consumption of wheat and its products is particularly high (approximately 135 kg per year according to FAO data) [6]. Additionally, the wheat production sector is of significant importance to the regional economy.

One of the main factors contributing to the decrease in wheat yield is the spread of airborne fungal diseases [7]. According to Savary et al. [8], fungal diseases cause the most significant losses among the 31 pests and pathogens reported in wheat.

Rust diseases (*Puccinia* spp.) are the most widespread and damaging wheat pathogens [9]. Globally, wheat yield losses caused by rust are estimated at 15 million tons per year [10].

In Central Asia, a wide range of virulent variants from the three main wheat rust diseases (yellow, stem, and leaf rust) is evolving [11], and their rapid spread and high genetic variability enable them to overcome previously effective sources of resistance, leading to the breakdown of widely cultivated varieties [12,13,14].

In Kazakhstan, rust outbreaks occur, on average, every 3–4 years due to favorable climatic conditions and high variability among pathogen populations [13,15,16,17]. In winter wheat-growing areas, particularly in the southern and southeastern regions, yellow (*Puccinia striiformis* f. sp. *tritici*) and leaf rusts (*Puccinia triticina*) are the main pathogens [14,17,18], and yield losses from yellow rust ranged from 7–10% in years of moderate development to 20–30% during severe outbreaks [14,17].

In the vast steppe regions of Northern Kazakhstan, where spring wheat is grown, stem (*P. graminis*) and leaf rusts (*P. triticina*) are predominant, and disease severity (DS) varies from 20 to 90%, depending on weather conditions during the growing season and the agroecological characteristics of the regions [13,17].

Currently, more than 235 genes with resistance to the three rust diseases have been identified in wheat and its wild relatives, but most confer disease-specific resistance [19]. In Kazakhstan, the *Yr5*, *Yr10*, and *Yr15* genes remain effective against yellow rust [14,18], while effective genes for leaf rust include *Lr9*, *Lr19*, *Lr34*, *Lr37*, and *Lr68* [17].

Common bunt (*Tilletia tritici* and *Tilletia laevis*), which leads to reduced grain quality and yield loss, is another highly damaging disease of winter wheat [20]. Infected plants produce dark spore-filled kernels instead of healthy grains, significantly reducing the crop food value [21], and yield losses can reach 20–50% under severe infection [22].

Since the 2000s, winter wheat areas affected by common bunt have increased in Kazakhstan and neighboring Central Asian countries [22,23], and disease severity depends on weather conditions, sowing practices, and the initial phytosanitary status of the seed material [24].

Resistance to common bunt is controlled by multiple *Bt* genes. To date, 16 resistance genes have been identified [19,25]. Among them, *Bt8*, *Bt9*, *Bt10*, *Bt11*, and *Bt12* are most commonly used in wheat breeding programs [22,26,27], and their effectiveness has been confirmed in Turkey, Iraq, and Kazakhstan [22,26,27,28]. However, the majority of registered wheat cultivars remain susceptible to common bunt, while the proportion of resistant varieties is still very low (0–4%) [26,29].

Under these conditions, genetic resistance remains the most effective, economically viable and environmentally safe approach to wheat protection [17,26]. Modern breeding combines traditional phenotypic selection with marker-assisted selection (MAS) to develop resistant cultivars. This approach enables molecular markers linked to resistance genes to be identified and utilized, thereby accelerating the development of resistant varieties with durable and complex resistance [30].

The Kazakh Research Institute of Agriculture and Plant Growing (KRIAPG) actively incorporates wild wheat relatives into winter wheat breeding using CIMMYT materials and international nurseries. This strategy enriches the genetic base with valuable resistance alleles [31,32]. In this context, utilizing a wide range of DNA markers to breed rust- and common bunt-resistant wheat is becoming increasingly important, as it allows lines carrying target alleles to be identified and accelerates the selection process.

Thus, the aim of this study is to integrate phenotypic and molecular approaches (MAS) to identify and select advanced winter wheat breeding lines resistant to common bunt and rust diseases.

## 2. Results

### 2.1. Field Evaluation of Adult Plant Resistance

#### 2.1.1. Yellow Rust

**Figure 1:**
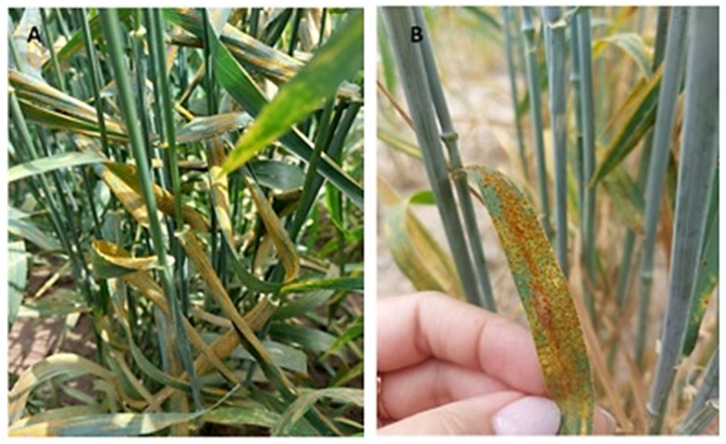
Symptoms of yellow (**A**) and leaf rusts (**B**) in advanced winter wheat breeding lines under artificial infection conditions (southeastern Kazakhstan).

Under the artificially created infection background, yellow rust development on the susceptible standard variety (Morocco) reached 60–100%, indicating high infection pressure, and this allowed us to reliably differentiate the studied breeding lines according to their resistance level (Figure 1).

**Figure 2:**
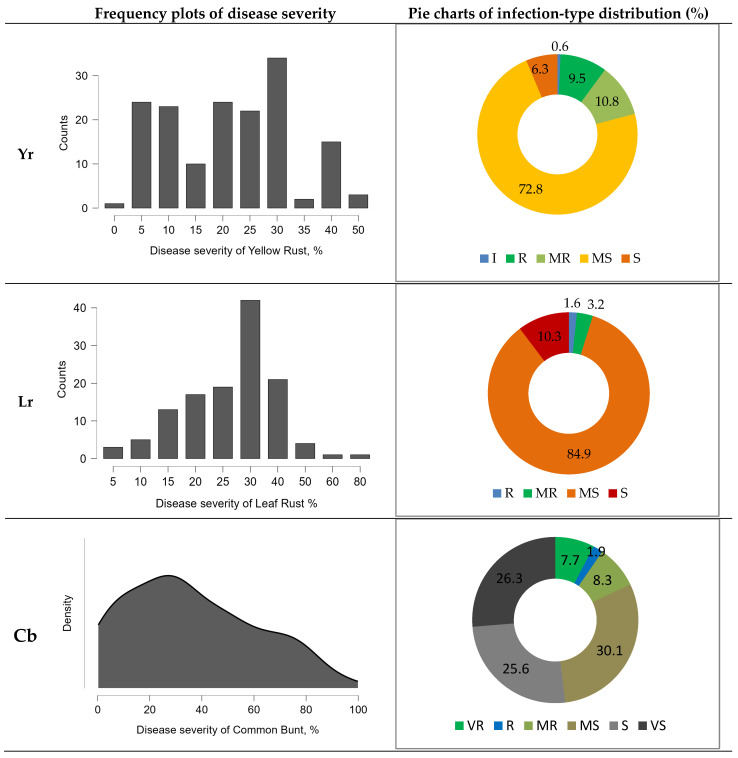
Resistance to yellow rust (Yr), leaf rust (Lr), and common bunt (Cb) under artificial infection conditions: frequency distribution of disease severity and percentage distribution of infection types—I: immune; VR: very resistant; R: resistant; MR: moderately resistant; MS: moderately susceptible; S: susceptible; and VS: very susceptible.

The studied genotypes showed significant variation in DS and reaction type (Figure 2). Among the 156 advanced breeding lines and five standard varieties, 10 genotypes were classified as susceptible (S) with 30–50% DS, and 118 genotypes were classified as moderately susceptible (MS) with 20–40% DS.

Seventeen genotypes were classified as moderately resistant (MR) with 10–20% DS, while 15 genotypes were resistant (R), with DS not exceeding 5%. One genotype (9909) showed no symptoms and was classified as immune (0) (Table S1, Supplementary Materials).

#### 2.1.2. Leaf Rust

Among the advanced breeding lines and standard varieties studied, 13 samples were classified as susceptible (S), while 107 were classified as moderately susceptible (MS) (Figure 2). Four genotypes showed a moderately resistant (MR) reaction, while two genotypes were resistant (R), with DS not exceeding 1–5% (Table S1, Supplementary Materials).

#### 2.1.3. Common Bunt

Common bunt severity on the susceptible standard variety Bogarnaya 56 ranged from 70 to 90%, indicating sufficient infection pressure under the artificial inoculation background.

The studied genotypes exhibited wide variation in DS and reaction type (Figure 2). Forty-one, forty, and 47 genotypes were classified as very susceptible (VS), susceptible (S), and moderately susceptible (MS), respectively, with 50–95.9%, 31–50%, and 11–30% DS (Table S1 of the Supplementary Materials).

Thirteen genotypes were classified as moderately resistant (MR), and three genotypes (9910, 18411-1-1, and 10/1013) were grouped as resistant (R) with a DS of 1–5%. Twelve genotypes (21266-3, K-1716-24, Sultan-2-2, 20061-12, 20114-16, 19980-6, 22382, 9908, 9909, 9926, 22414-1, and 22414-2) showed no symptoms and were classified as very resistant (VR), maintaining low DS under high infection pressure.

### 2.2. Genotyping of Rust Resistance Genes

#### 2.2.1. Genotyping of Yellow Rust Resistance Genes

In the present study, we identified three yellow rust resistance genes (*Yr5*, *Yr10*, and *Yr15*) that were considered effective in regions of Central Asia and Kazakhstan [18].

Two STS markers (S19M93 [33] and S23M41 [33]) were used to identify the genotypes carrying the *Yr5* resistance allele. An isogenic line, №7-20ENTRY, from the differential set 20RUST-DIF-SET, carrying the *Yr5* resistance gene, was used as a positive control. The S19M93 marker allowed us to identify 59 out of the 161 studied winter wheat samples that produced a 100 bp amplification fragment (Figure S1, Supplementary Materials) identical to that of the *Yr5* positive control. According to a previous report, this fragment is associated with the *Yr5* resistance allele [33].

The second marker, S23M41, also allowed us to identify an amplification of a 275 bp fragment in 59 out of 161 winter wheat samples, which, according to Smith [33], is associated with the *Yr5* resistance gene allele (Figure S2, Supplementary Materials).

The concordance results of the two markers revealed 58 carriers of the *Yr5* resistance allele, including 33 advanced breeding lines from the CVT nursery, 21 lines from the CTN, and 4 standard cultivars (Table 1).

To identify the carriers of the *Yr10* resistance gene alleles, two markers were used: the SCAR marker Yr10SCAR [34] and the SSR marker psp3000 [34,35]. An isogenic line, №12-20ENTRY, carrying the *Yr10* resistance gene from the differential set 20RUST-DIF-SET was used as a positive control.

The Yr10SCAR marker allowed us to identify 42 winter wheat samples with amplification of a 200 bp band (Figure S3, Supplementary Materials), which is associated with the *Yr10* resistance allele [23], and a 180 bp fragment was detected in the remaining lines.

For *Yr10*, we only identified nine samples that produced a 286 bp band, corresponding to the band size of the *Yr10* control line, from the second marker, psp3000 (Figure S4, Supplementary Materials), and fragments of 240 bp and 220 bp were amplified in the remaining samples.

Based on the concordance of the two DNA markers, only one line (952) from the CVT breeding nursery carried the allele associated with *Yr10* resistance (Table 1).

To identify carriers of the *Yr15* gene alleles, two diagnostic SSR markers, gwm413 and barc8 [36,37], were used. The isogenic *Yr15* line (№13-20ENTRY) was used as a positive control, and the gwm413 marker allowed us to identify 15 winter wheat samples with a 96 bp band (Figure S5, Supplementary Materials) identical to that of the positive control.

However, based on the PCR results of the second marker (barc8), we only observed five lines with a 200 bp band that corresponds to the fragment size of the *Yr15* control line (Figure S6, Supplementary Materials). In the remaining lines, 250 bp and 260 bp fragments were amplified.

The PCR results obtained with the gwm413 and barc8 markers were not concordant (Table S1, Supplementary Materials). Thus, it was concluded that reliable *Yr15* gene carriers were absent among the studied winter wheat breeding lines.

#### 2.2.2. Genotyping of Leaf Rust Resistance Genes and Pleiotropic Rust Resistance Loci

PCR analysis using the SCS5_550_ marker was performed to identify carriers of the *Lr9* gene [38]. The wheat cv. Phyton *Lr9* was used as a positive control, and the results of the analysis showed that amplification of the expected fragment (550 bp) was found in six (3.7%) samples, including two advanced breeding lines from the CVT nursery, three lines from the CTN, and one standard variety (Figure S7, Supplementary Materials).

The pleiotropic rust resistance loci *Lr34*/*Yr18*/*Sr57* were identified using the STS marker csLV34 [39], and the NIL-Thatcher-Lr34-PI58548 (RL6058) line was used as a positive control. According to the results, the presence of a 150 bp fragment associated with the *Lr34*/*Yr18*/*Sr57*/*Pm38* gene resistance allele was detected in 80 samples (49.7%), comprising 41 advanced breeding lines from the CVT nursery, 35 lines from the CTN, and four standard varieties. A 229 bp amplicon indicating the absence of the gene was found in the remaining lines (Figure S8, Supplementary Materials).

DNA identification of the *Lr37*/*SR38*/*Yr17* gene complex was performed using the Ventriup-F and LN-2-R primers [40]. Two near-isogenic lines, NIL-THATCHER-Lr37-VPM (RL6081), which carries *Lr37*, and Trident-Sr38, which carries *Sr38*, were used as positive controls. According to the PCR results, the presence of the 259 bp fragment desired was recorded in 40 (24.8%) winter wheat breeding lines (Figure S9, Supplementary Materials).

Based on genotyping of 156 winter wheat breeding lines and five standard varieties, PCR products associated with the resistance alleles of the *Yr5*, *Yr10*, *Lr9*, *Lr34*/*Yr18*/*Sr57*, and *Lr37*/*Sr38*/*Yr17* genes were observed in 58, one, six, 80, and 40 breeding lines, respectively (Table 1).

### 2.3. Genotyping of Common Bunt Resistance Genes

In this study, the DNA identification of 156 advanced breeding lines and five standard winter wheat varieties was performed using five common bunt resistance genes (*Bt8*, *Bt9*, *Bt10*, *Bt11*, and *Bt12*).

The *Bt8* and *Bt11* genes were identified using the gwm114 marker [41]. This marker allows us to simultaneously identify two 180 bp and 120 bp fragments (Figure S10, Supplementary Materials), which correspond to the *Bt8* and *Bt11* genes located on the 3B chromosome [23,42], and isogenic wheat lines M78-9496 (*Bt8*) and M82-2123 (*Bt11*) were used as positive controls to verify the PCR identification results. We also identified 42 samples with a 180 bp band that is identical to that of positive control M78-9496 using the gwm114 marker. In addition, 145 samples showed a 120 bp corresponding to that of the M82-2123 line (Table 2).

The marker gpw7433 was used to identify carriers of the *Bt9* gene, which is located on chromosome 6D [43]. The expected fragment size of 296 bp, which is identical to that of the positive control M77-1140 (*Bt9*), was detected in 126 (78.3%) winter wheat samples (Table 2 and Figure S11, Supplementary Materials).

Additionally, the FSD/RSA marker, which was identified based on SCAR and located on the 6D chromosome, was used in *Bt10* detection [44]. An isogenic wheat line, M83-1621 (*Bt10*), was used as a positive control. In our study, PCR analysis with this marker revealed a 275 bp diagnostic fragment associated with a *Bt10* resistance allele in 67 (41.6%) winter wheat samples (Table 2).

The markers gwm264 [45] and gwm374 [45], which are linked to the *Bt12* resistance gene, were used for the molecular detection of this gene. The 190 bp band characteristic of the Xgwm264 marker was found in 113 lines (Figure S12, Supplementary Materials), while the expected 180 bp band for the gwm374 marker was amplified in 146 wheat samples (Figure S13, Supplementary Materials). Concordance between the two markers was observed in 103 (64%) breeding lines and standard varieties (Table 2).

### 2.4. SY Evaluation Under Natural Field Conditions

The natural field background was used to evaluate the productivity of the studied winter wheat breeding lines and to select high-yielding genotypes. The yield of the advanced breeding lines in the CVT nursery ranged from 2.9 to 6.0 t/ha, with an average value of 4.1 t/ha. In the CTN, yields ranged from 3.1 to 6.5 t/ha, with an average value of 4.3 t/ha. Additionally, the yield of the standard varieties was 4.8–5.0 t/ha, with an average value of 4.8 t/ha (Table 3).

Eleven advanced breeding lines (K-2041-13-1, D302, D304, D305, D306, D307, 9828, 9825, 9813, 9807, and 9926) have been identified, exceeding the average yield of five standard varieties (4.8 t/ha) by 10–15%.

### 2.5. Comparison of Disease Resistance Phenotypes and Genotyping Data

The genotyping results on disease resistance were compared with the phytopathological analysis data to determine the practical agronomic value of the studied breeding lines.

Five advanced breeding lines (9829, 9910, 9909, D305, and 21589-12), in which two or three resistance alleles associated with yellow rust resistance were identified using DNA markers and which exhibited high resistance (0I–5R) under an infection background, were selected (Table 4).

Two advanced breeding lines (9910 and 9909) carrying two or three resistance alleles associated with leaf rust, as identified using DNA markers, were selected. Both lines showed a resistance reaction of 10MR under an infection background (Table 4).

Fourteen advanced breeding lines (19251-2, 20232-14, 20841-2, 20841-17, D68, 20061-12, 10/1013, 20437-9, K-1716-24, 20114-16, 22416, 22382, 9908, and 9909) carrying three to five alleles associated with common bunt resistance were identified. These lines exhibited low disease severity (DS ≤ 10%) under infection conditions.

Based on the analysis of two breeding nurseries, two unique winter wheat lines, 9909 (*Lr34*/*Yr18*/*Sr57*, *Lr37*/*SR38*/*Yr17*, *Bt9*, *Bt11*, and *Bt12*) and 9910 (*Yr5*, *Lr34*/*Yr18*/*Sr57*, *Lr37*/*SR38*/*Yr17*, *Bt9*, and *Bt11*), were identified. These lines combined multiple resistance alleles detected by DNA markers and showed resistance to yellow rust and common bunt. The 9909 and 9910 lines were also characterized by high productivity, with yields of 4.9 and 5 t/ha, respectively, exceeding the yields of the standard varieties by 2–4%.

Among the advanced breeding lines selected based on field resistance to yellow rust and the presence of a valuable gene complex, the D305 line (*Yr5*, *Yr17*/*Lr37*, *Bt9*, *Bt11*, and *Bt12*) also showed high productivity, and its yield reached 5.4 t/ha, which is 11% higher than the average yield of the standard varieties (4.8 t/ha).

Statistical analysis using the Mann–Whitney U test was performed to compare DS (percentage of infected plants) between groups of samples carrying and not carrying alleles associated with resistance genes (*Yr5*, *Yr10*, *Yr15*, *Lr9*, *Lr34*/*Yr18*, *Lr37*/*Yr17*, *Bt8*, *Bt9*, *Bt10*, *Bt11*, and *Bt12*), as determined via genotyping with 14 DNA markers.

**Figure 3:**
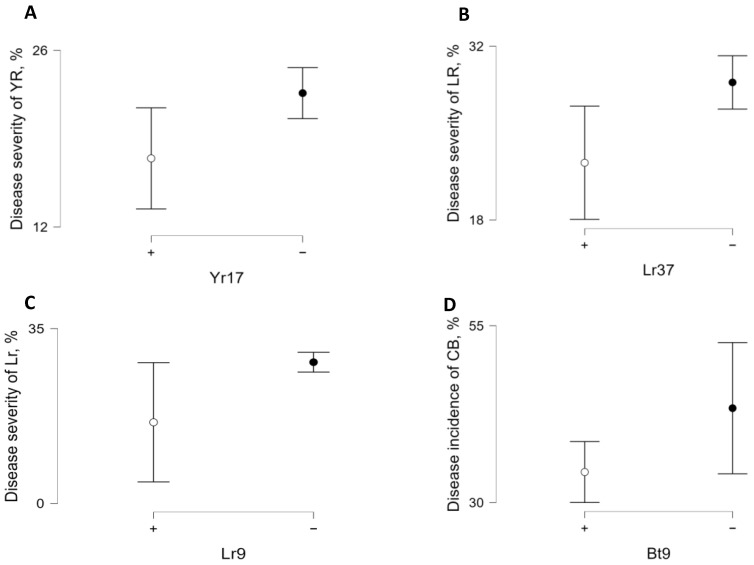
Disease severity distributions for yellow rust (Yr), leaf rust (Lr), and common bunt (Cb) in winter wheat lines according to the presence of resistance alleles detected using the molecular markers Ventriup-LN2 (*Lr37*/*Yr17*) (**A**,**B**), SCS5550 (*Lr9*) (**C**), and gpw7433 (*Bt9*) (**D**). Differences between groups were evaluated using the Mann–Whitney U test (White (open) circle — represents the mean value for one group, Black (filled) circle — represents the mean value for the other group).

Significant differences in DS were only observed for three markers: Ventriup-F/LN-2-R (associated with the *Lr37*/*Yr17* locus), SCS5_550_ (associated with *Lr9*), and Xgpw7433 (associated with *Bt9*) (Figure 3 and Table S2, Supplementary Materials).

In particular, lines carrying the marker allele associated with the gene complex *Lr37*/*Yr17* showed significantly lower DS for both yellow rust and leaf rust compared to lines lacking this gene complex (Figure 3), suggesting a potential association between the marker and reduced DS.

Based on the analysis of wheat breeding lines with combinations of resistance-associated alleles, we identified nine groups with yellow rust resistance, seven groups with leaf rust resistance, and 25 groups with common bunt resistance, and the allele combinations are presented in Table S1.

To evaluate the relationship between allele combinations and infection type (IT) under infection conditions, a Chi-square test (χ^2^) was performed (Table S3, Supplementary Materials).

No statistically significant association was found for any of the diseases studied. Specifically, allele combinations were not significantly associated with resistance categories for common bunt (χ^2^ = 108.7; *p* < 0.76), yellow rust (χ^2^ = 32.3; *p* < 0.45) and leaf rust (χ^2^ = 23.7; *p* < 0.32). These results indicate that, in the breeding material studied, allele combinations did not significantly influence the phenotypic resistance measured by IT under the given environmental and pathogenic conditions.

## 3. Discussion

### 3.1. Integrated Phenotypic and Molecular Approaches in Wheat Breeding

Modern breeding focuses on a rapid response to abiotic and biotic stresses and on the development of new-generation cultivars with high adaptive capacity and genetic resistance [46,47]. Using resistant varieties is both economically and agronomically sound, especially under recurrent outbreaks of rust and bunt diseases in traditional wheat-growing regions [48,49]. An integrated approach combining marker-assisted selection (MAS) with phenotypic evaluation under specialized infection backgrounds can improve selection accuracy and accelerate the development of resistant cultivars [17,26,27,49]. In this study, this approach enabled genotypes in southeastern Kazakhstan with reduced disease severity and putative resistance to yellow rust, leaf rust, and common bunt to be identified.

### 3.2. Phenotypic Evaluation of Resistance Under Infection Conditions

The use of infection nurseries minimizes the influence of weather conditions and uneven natural infection, and this is essential to reliably differentiate genotypes by resistance level [50,51]. In our experiments, disease development was more rapid during the 2024 humid growing season, whereas it progressed more slowly during the drier seasons with lower precipitation (2023 and 2025). Nevertheless, the high DS (70–90%) observed in susceptible standards indicates sufficient infection pressure and reduces the likelihood of a “disease escape” effect. This supports the reliability of the phenotypic evaluation and suggests that the selected genotypes express true field resistance rather than apparent infection avoidance due to unfavorable environmental conditions or an uneven infection background.

Out of the 156 advanced winter wheat breeding lines classified as resistant (R/VR), 15 (9.6%), two (1.3%), and 15 (9.6%) genotypes were resistant to yellow rust, leaf rust, and common bunt, respectively. The low DS values (0–5%) under high disease pressure in susceptible standards indicate stable field resistance across the study years, which included both dry (2023 and 2025) and humid (2024) seasons. Additionally, the overall proportion of resistant genotypes in the breeding material was low.

Based on a comparison of the published data, we found that the frequency of resistant genotypes observed in this study is consistent with trends reported in other regions under artificial infection. Specifically, Aydoğdu M. [29] reported no resistant varieties to common bunt among 30 wheat samples under infection conditions in Turkey. However, in another study from the Antalya region, only two (1.9%) out of 102 varieties were resistant, whereas 72.5% were susceptible [26], and under infection conditions in the Almaty region of Kazakhstan, only seven (10%) out of 60 local and foreign wheat varieties demonstrated resistance over three years [23].

For yellow rust, our results are also in agreement with previous studies. In India, only 3.6% of the 110 wheat genotypes were classified as immune under artificial inoculation with *Puccinia striiformis* f. sp. *tritici*. In addition, 6.3% of genotypes demonstrated slow-rusting resistance [52]. In the Russian Federation, an evaluation of 72 registered winter wheat varieties (2019–2022) revealed that only 13 cultivars (18%) were resistant to five yellow rust isolates [53], and in Kazakhstan, only 11 (15.7%) out of 70 winter wheat genotypes were resistant to yellow rust [18].

In terms of leaf rust resistance, only nine out of 42 Egyptian wheat varieties were resistant at both the seedling and adult plant stages during two growing seasons [54]. Similarly, in Ethiopia, only 58 (3.8%) out of 1500 samples showed resistance to leaf rust [55]. These findings support the conclusion that resistance to yellow rust, leaf rust, and common bunt occurs at low frequencies in both breeding and commercial wheat varieties. This highlights the need for continued and more targeted identification of resistance sources and the incorporation of effective resistance genes into modern breeding programs.

Unlike phenotypic evaluation, molecular marker-based approaches enable valuable resistance alleles to be detected regardless of the plant development stage and weather conditions. Moreover, they also allow genotypes carrying multiple resistance genes to be detected simultaneously [53,56,57].

### 3.3. Molecular Characterization and Marker–Trait Associations in Relation to Disease Resistance

In our study, screening of Kazakhstani winter wheat breeding materials revealed that 58 (36%) samples carried the *Yr5* resistance gene allele, while one sample (0.6%) carried the *Yr10* resistance gene allele, and previous studies have also reported the presence of these genes in Kazakhstani germplasm [18].

Notably, the results obtained with two markers (gwm413 and barc8) for *Yr15* detection were inconsistent, which prevents the reliable identification of the *Yr15* resistance allele in the studied material. Such discrepancies may be attributed to the inherent limitations of DNA marker-based approaches, including recombination events, allelic variation, and differences in marker efficiency, which can lead to false-negative or ambiguous results [56,57]. Several studies have shown that the *Yr15* gene remains rare and is mainly found in specialized germplasm [30,53,58]. This suggests potential gaps in Kazakhstani wheat breeding programs and highlights the importance of further introgression of *Yr15* while also considering the limitations of MAS.

We identified 80 (49.7%) lines carrying a fragment associated with the *Lr34*/*Yr18*/*Sr57* gene resistance allele and 40 (24.8%) lines carrying a fragment linked to the *Lr37*/*Yr17*/*Sr38* resistance gene complex. The pleiotropic *Lr34*/*Yr18*/*Sr57* gene is known to confer durable, broad-spectrum resistance to leaf, yellow, and stem rusts at the adult plant stage. It is widely recognized as a “slow-rusting” gene and encodes an ABC transporter that enhances cell wall reinforcement and provides protection against multiple fungal pathogens [59]. The *Lr37*/*Yr17*/*SR38* gene complex originates from a 2NS/2AS chromosomal translocation introgressed from *T. ventricosum* into wheat [18]. These genes are widely distributed among wheat cultivars and are commonly used in modern breeding programs, including those in Kazakhstan and neighboring regions [17,18,53,58].

It should also be noted that, in addition to race-specific resistance genes, adult plant resistance (APR) genes such as *Lr34*/*Yr18* contribute to quantitative, non-race-specific resistance that is generally more stable across environments. However, the contribution of APR genes was not quantitatively evaluated in this study, which may partly explain the variability observed in phenotypic responses among genotypes.

The *Lr9* gene, a race-specific resistance gene transferred into *Triticum aestivum* from *Aegilops umbellulata*, was detected in only six (3.7%) wheat samples. Previous studies have also reported low frequencies of this gene (10–12%) or even its absence in some germplasm collections [60,61,62].

In this study, common bunt resistance genes (*Bt9*, *Bt11*, and *Bt12*) with frequencies of 78.3%, 90%, and 64%, respectively, were detected. In contrast, the *Bt8* and *Bt10* genes were less frequent, at 26.1% and 41.6%, respectively, and similar patterns have been reported in other studies, including those on Kazakhstani wheat cultivars [23,26].

A particularly important aspect of this study was the comparison of DNA-based marker data with phenotypic evaluation under artificial infection. Despite the presence of resistance-associated *Bt* gene alleles, most genotypes showed moderate DS (20–40%). Consequently, it should be noted that marker presence does not necessarily indicate phenotypic resistance.

The Mann–Whitney U test revealed that breeding lines carrying the *Bt9*-associated marker had significantly lower levels of infection compared to those lacking this marker under conditions in southeastern Kazakhstan. This indicates that *Bt9* contributes to enhanced resistance to common bunt in the tested material. In contrast, no statistically significant association was detected for other *Bt*-associated markers, suggesting that their effects may be limited, background-dependent, or influenced by environmental conditions and pathogen variability, and similar findings have been reported previously [63], suggesting the limited effectiveness of individual resistance genes. This may be explained by the high virulence of local pathogen populations capable of overcoming specific resistance genes.

At the same time, 14 breeding lines (19251-2, 20232-14, 20841-2, 20841-17, D68, 20061-12, 10/1013, 20437-9, K-1716-24, 20114-16, 22416, 22382, 9908, and 9909) carrying combinations of *Bt*-associated markers showed high resistance (DS of 0–5%). These genotypes represent valuable sources of resistance and can be used as promising donors to develop winter wheat cultivars. However, further validation under diverse environmental conditions is required to confirm the stability and effectiveness of this resistance.

A statistically significant difference in terms of disease severity was also observed between breeding lines carrying markers associated with *Lr9* and *Lr37*/*Yr17*/*Sr38*, as well as those lacking these markers. These results indicate that the presence of these loci contributes to reduced infection levels under conditions in southeastern Kazakhstan. At the same time, the detection of significant effects in only a subset of markers highlights the importance of validating marker–trait associations within specific genetic backgrounds and environments.

In contrast, for yellow rust and leaf rust, no significant association was observed between disease severity and resistance-associated markers in wheat samples. This discrepancy may be explained by the biological features of the “wheat–*Puccinia striiformis*” pathosystem, as many *Yr* genes are race-specific, and their effectiveness largely depends on the virulence structure of local pathogen populations. Although pathogen diversity in the region has been previously reported [14], the population structure was not directly assessed in this study, which limits the ability to link resistance effectiveness with specific virulence profiles.

The infection background in the southern and southeastern regions of Kazakhstan is characterized by high genetic and racial diversity of *Puccinia striiformis*, due to favorable overwintering conditions and an extended vegetation period [14]. Under such conditions, pathogen populations may contain races capable of overcoming the resistance conferred by individual genes. As a result, the presence of resistance alleles does not always lead to an immune response in plants.

The infection background in southern and southeastern Kazakhstan is characterized by *Puccinia striiformis* with high genetic and racial diversity, and this region is associated with favorable overwintering conditions and an extended vegetation period [14]. Under such conditions, pathogen populations may contain strains capable of overcoming the resistance conferred by individual genes, thus explaining why the presence of resistance-associated markers does not always lead to a decrease in DS.

Environmental factors also play a significant role. During the study period (2023–2025), the average temperatures in May and June exceeded long-term average values by 2–3 °C. Since many *Yr* genes are temperature-sensitive, their effectiveness may decrease at temperatures above 18–25 °C [64], contributing to discrepancies between genotypic and phenotypic data. This highlights the importance of genotype × environment interactions in resistance expression.

It should also be noted that DNA markers do not always confirm the presence of functional resistance genes, and the absence of phenotypic effects despite the presence of resistance-associated markers may reflect recombination events, allelic variation, or the influence of the genetic background [56], as well as other factors such as epistatic interactions, incomplete penetrance, and limitations in marker specificity and validation [57]. Therefore, marker-based results should be interpreted with caution and validated through phenotypic evaluation under diverse environmental conditions.

The analysis of combinations of resistance-associated alleles revealed relatively high diversity among the genotypic groups (nine groups for yellow rust, seven for leaf rust, and 25 for common bunt). However, no statistically significant associations were detected between allele combinations and disease response. This observation can be attributed to the large number of genotype combinations, which may have reduced sample sizes within individual groups and limited the statistical power of the analysis. In addition, resistance expression is likely influenced by interactions among multiple genes, genetic backgrounds, and environmental conditions, which may mask the effects of individual allele combinations.

Overall, our results indicate that resistance is controlled by complex GxE interactions rather than by individual genes alone.

### 3.4. Identification of Promising Breeding Lines with Field Resistance Using Molecular Markers

Based on a comparison of molecular marker data with field resistance evaluation, we found that the most promising genotypes combined resistance alleles with low DS under an infection background. In particular, lines 9910 (*Yr5*, *Lr37*/*Sr38*/*Yr17*, *Lr34*/*Yr18*/*Sr57*, *Lr9*) and 9909 (*Lr37*/*Sr38*/*Yr17*, *Lr34*/*Yr18*/*Sr57*) demonstrated high resistance to yellow rust (0I–5R) and moderate resistance to leaf rust (10MR).

Line 9909 (*Lr37*/*Sr38*/*Yr17*, *Lr34*/*Yr18*/*Sr57*, *Bt9*/*Bt11*/*Bt12*) was also identified as a particularly valuable genotype based on a comprehensive evaluation, showing high resistance to yellow rust (0I), leaf rust (10MR), and common bunt (VR) under artificial infection.

Grain yield was included as an important complementary trait under field conditions, as prospective cultivars should combine disease resistance with competitive productivity. The selected winter wheat breeding lines (9910, 9909, and D305) showed yields comparable to those of the standard or exceeded it by 4–11%. Although these differences are moderate, such gains are considered agronomically meaningful, especially when combined with stable disease resistance.

These results highlight the importance of integrating molecular marker analysis with phenotypic evaluation in wheat breeding programs, as phytopathological assessment alone cannot identify hidden carriers of resistance genes at early breeding stages, whereas using molecular markers alone does not guarantee effective field resistance.

Therefore, an integrated approach that combines DNA-based screening with mandatory evaluation under artificial infection conditions is the most efficient strategy. This is particularly important in regions with high pathogen variability, such as southern Kazakhstan, where the effectiveness of resistance genes depends on local pathogen populations and environmental conditions.

## 4. Materials and Methods

### 4.1. Plant Material

The study material comprised 156 advanced winter wheat breeding lines developed at the Laboratory of Cereal Crop Breeding of KRIAPG. These lines originated from two breeding nurseries: the control testing nursery (CTN), comprising 86 lines, and the competitive variety testing (CVT), comprising 70 lines. Local wheat varieties (Egemen-20, Almaly, Zhetysu, Steklovidnaya-24, and Talimi-80) were used as standard reference varieties.

For PCR identification of resistance genes, near-isogenic wheat lines carrying the genes *Yr5*, *Yr10*, *Yr15*, *Lr9*, *Lr34*, *Lr37*, *Bt8*, *Bt9*, *Bt10*, *Bt11*, and *Bt12* were used as positive controls, and these materials were obtained from the Laboratory of Immunity and Plant Protection (CIMMYT–Turkey materials).

### 4.2. Field Experiments and SY Evaluations

Field experiments were conducted during the 2023–2024 and 2024–2025 growing seasons at the experimental field station of the Cereal Crop Breeding Laboratory of KRIAPG. The experimental site is located in the foothill zone of southeastern Kazakhstan (Almaty region) at an altitude of 740 m above sea level (43°13′38.4″ N 76°42′08.2″ E), and the breeding lines were grown under rainfed conditions.

Winter wheat was sown at the optimal time for the region, that is, during the first ten days of October, and the breeding lines were planted on 15 m^2^ plots at a seeding rate of 0.18 kg/m^2^. The CTN lines were evaluated in a single replication due to the large number of entries and limited seed availability, following the standard layout of such trials. In contrast, the CVT lines were evaluated in a randomized design with three replications to ensure higher statistical accuracy and the reliability of the results.

At full grain maturity, yield was evaluated according to the Methodology of the State Variety Testing of Agricultural Crops [65], and plots were harvested at 12% moisture using a Classic Plus plot combine (Wintersteiger, Ried im Innkreis, Austria). The average yield of the studied wheat lines was calculated based on data from two growing seasons.

### 4.3. Assessment of Field Response to Fungal Diseases

During the 2022–2023, 2023–2024, and 2024–2025 growing seasons, winter wheat breeding lines were also evaluated in a specialized infection nursery of the Laboratory of Immunity and Plant Protection (43°14′49.0″ N 76°42′05.0″ E).

The experimental site is located in an agroecological zone of Kazakhstan with a high natural prevalence of the studied diseases (*Puccinia striiformis*, *Puccinia triticina*, *Tilletia tritici*, and *Tilletia laevis*). This location is characterized by stable pathogen pressure and includes a specialized experimental field designed for resistance evaluation under controlled infection conditions.

Winter wheat samples were sown in single-row plots (1 m length) with 30 seeds per row and 20 cm spacing between rows, and sowing was carried out in early October. Additionally, the experiments were conducted in a randomized design with three replications.

To ensure uniform infection, spreader rows were included and sown every 20 rows. The susceptible cultivar Morocco (Morocco, North Africa) was used as a spreader for leaf and yellow rusts, while the susceptible cultivar Bogarnaya 56 (Kazakhstan) was used for common bunt.

Resistance to rust (*Puccinia* spp.) and bunt (*Tilletia* spp.) pathogens was evaluated under artificial infection conditions over three growing seasons, and disease severity (DS) and infection type (IT) were recorded annually. For the final assessment, the maximum value observed across years was used.

Artificial inoculation with common bunt (*Tilletia* spp.) was performed two days before sowing by treating wheat seeds with spores of the local pathogen population. Seeds and teliospores were mixed at a ratio of 1:100 and shaken manually until an even coating was achieved, and this method is effective for inoculating small seed samples [66].

DS was expressed as the percentage of infected spikes within each row, and DS for common bunt was assessed at the waxy ripeness stage and calculated as a portion of infected spikes using the following formula:

DS = number of infected spikes/total number of spikes × 100(1)

Genotypes containing 0% infected spikes were classified as very resistant (VR) to common bunt, while those containing 1–5% infected spikes were considered resistant (R). Infection levels of 6–10%, 11–30%, 31–50%, and 51–100% corresponded to moderately resistant (MR), moderately susceptible (MS), susceptible (S), and very susceptible (VS), respectively [26,67].

For rust diseases, artificial inoculation was carried out during the tillering–booting stages using a mixture of *P. striiformis* and *P. triticina* urediniospores with talc powder (1:100) at a rate of 20 mg spores/m^2^ [68], and disease assessments began from the moment visible symptoms appear and were repeated at 10-day intervals until the milky–waxy ripeness stage.

IT and DS (%) were used to assess resistance. According to the CIMMYT scale [69,70], IT was categorized as follows: 0 (immune)—no symptoms; R (resistant)—small necrotic areas without pustules; MR (moderately resistant)—small pustules surrounded by chlorotic and necrotic zones; MS (moderately susceptible)—medium-sized pustules with chlorosis but without necrosis; and S (susceptible)—large pustules without chlorosis or necrosis.

DS (%) was determined using the modified Cobb scale [71].

### 4.4. Meteorological Conditions During the Study Period

According to the Köppen climate classification [72], the climate of the Almaty region is Dfa (continental with hot summers), and the experimental site is characterized by light chestnut soils with low humus content (1.6–1.9%), slight alkalinity (pH 7.8), and a clay content of up to 34.9% [73].

Meteorological data, including precipitation and average air temperature, were recorded during the study period using an automatic weather station (iMETOS (IMT300USW, Pessl Instruments, Weiz, Austria)). The weather station (43°14′06.4″ N 76°41′25.2″ E) was located 1300 m from the wheat breeding nursery and 1500 m from the specialized infection nursery, ensuring representative environmental conditions for both experimental sites. Monthly precipitation and air temperature data for the winter wheat growing period are presented in Table S4 (Supplementary Materials).

Total precipitation during the spring period (April–May) was 68.2–43.4 mm in 2023, 111.3–121.1 mm in 2024, and 57.2–80.4 mm in 2025, while summer precipitation (June–July) ranged from 4.3 to 33.6 mm in 2023, from 19.7 to 85.2 mm in 2024, and from 9.6 to 17.1 mm in 2025. Compared with the long-term average (65.7–110.6 mm for 1991–2023), the spring of 2024 was humid, whereas 2023 and 2025 were relatively dry.

The average monthly air temperatures during spring (April–May) ranged from 11.9 to 17.2 °C (2023), from 12.8 to 17.6 °C (2024), and from 15.7 to 20.8 °C (2025) compared with the long-term average of 10.9–16.3 °C. However, in June, temperatures exceeded the long-term average by 3.2–4.4 °C in all study years.

### 4.5. Molecular Markers of Resistance Genes

Genomic DNA was extracted from 7–10-day-old wheat seedlings grown in a greenhouse using the Dellaporta method [74]. DNA was isolated from 156 breeding lines and 10 standard cultivars, and specific SSR- and SCAR-based molecular markers were used to screen for the *Yr*, *Lr*, and *Bt* genes in winter wheat breeding lines from two nurseries.

Primers sequences and PCR conditions are provided in Table S5 (Supplementary Materials). Oligonucleotides were synthesized by Thermo Fisher Scientific (Pleasanton, CA, USA).

PCR was performed in a 20 μL reaction containing 2 µL of genomic DNA (100 ng), 2 µL of 10X PCR buffer, 2 µL of MgCI_2_ (2.5 mM), 1 µL of the dNTP mix (2.5 mM, each), 0.5 µL of each primer (1 pM/µL), 1 µL of DMSO (3%), and 0.3 µL of Taq polymerase (5 units/µL; Biosan, Novosibirsk, Russia). Conversely, amplification was carried out using an Eppendorf MasterCycler (Berzdorf, Germany).

PCR products were separated on 8% polyacrylamide gels (AppliChem, Darmstadt, Germany for 70 min at 200 V using an electrophoretic chamber, and gels were stained with ethidium bromide and visualized under UV light using a Quantum ST4 transilluminator (Vilber-Lourmat, Marne-la-Vallée, France), with a 50 bp DNA ladder (Step50, BiolabMix, Novosibirsk, Russia) used as a reference.

### 4.6. Statistical Analysis

Statistical analyses were performed using JASP version 0.95.4 [75], and descriptive statistics were calculated for SY.

To compare disease severity between groups differing in the presence or absence of resistance-associated alleles, the nonparametric Mann–Whitney U test was used. This test was chosen because the data were not normally distributed and the groups were independent. It is also suitable for percentage data, which often shows abnormal distributions and heterogeneous variance, and statistical significance was set at *p* < 0.05.

The Mann–Whitney U test was also used to evaluate whether the presence of resistance-associated alleles was associated with a reduced DS score.

To evaluate the association between combinations of resistance-associated alleles and infection type (IT), the Chi-square (χ^2^) test of independence was applied. This test was used to assess the relationships between the allele combination group and resistance categories, and statistical significance for the χ^2^ test was also set at *p* < 0.05.

## 5. Conclusions

The comparison of phenotypic data with molecular genotyping results revealed several unique lines among the 156 advanced winter wheat lines with resistance to the major rust diseases and common bunt:

- Five lines (9829, 9910, 9909, D305, and 21589-12) carrying valuable *Yr* genes demonstrated high resistance to yellow rust (0I-5R);
- Fourteen lines (19251-2, 20232-14, 20841-2, 20841-17, D68, 20061-12, 10/1013, 20437-9, K-1716-24, 20114-16, 22416, 22382, 9908, and 9909) carrying combinations of *Bt* genes demonstrated high resistance (DS 0–5%);
- Two advanced lines (9910 and 9909) carrying combinations of valuable rust resistance genes (*Yr5*, *Lr37*/*Yr17*, *Lr34*/*Yr18*, and *Lr9* and *Lr37*/*Yr17*, and *Lr34*/*Yr18*, respectively) exhibited high resistance to yellow rust (5R and 0I) and moderate resistance to leaf rust (10MR);
- A unique breeding line (9909) with several resistance genes (*Lr37*/*Yr17*, *Lr34*/*Yr18*, *Bt9*, *Bt11*, and *Bt12*) showed resistance to yellow rust (0I), leaf rust (10MR), and common bunt (VR) under artificial infection.

For the southern and southeastern regions of Kazakhstan, our results support the use of MAS targeting the *Lr9*, *Lr37*/*Yr17*, and *Bt9* genes as an effective approach to accelerate the development of wheat lines with improved resistance to rust diseases and common bunt. The observed associations between these markers and lower disease severity indicate the practical relevance of selected breeding lines under the studied environmental conditions. However, variation in marker effects suggests that their performance depends on genetic background. Therefore, these markers should be further validated across diverse breeding materials and pathogen populations to ensure their consistent effectiveness in breeding programs.

## Figures and Tables

**Table 1:** Number of winter wheat breeding lines carrying valuable alleles of the rust resistance genes.

Name of the Nursery	Total Samples	*Yr5*	*Yr10*	*Lr9*	*Lr34*/*Yr18*/ *Sr57*	*Lr37*/*Sr38*/ *Yr17*
CVT	86	33 (38.4%)	1 (1.2%)	2 (2.3%)	41 (47.7%)	15 (17.4%)
CTN	70	21 (30%)	0 (0%)	3 (4.3%)	35 (50%)	21 (30%)
Standard varieties	5	4 (80%)	0 (0%)	1 (20%)	4 (80%)	4 (80%)
Total	161	58 (36%)	1 (0.6%)	6 (3.7%)	80 (49.7%)	40 (24.8%)

**Table 2:** Number of winter wheat breeding lines carrying valuable resistance alleles of *Bt* genes.

Name of the Nursery	Total Samples	*Bt8*	*Bt9*	*Bt10*	*Bt11*	*Bt12*
CVT	86	38 (44.2%)	69 (80.2%)	42 (48.8%)	75 (87.2%)	58 (67.4%)
CTN	70	0 (0%)	52 (74.3%)	22 (31.4)	65 (92.9%)	44 (62.9%)
Standard varieties	5	4 (80%)	5 (100%)	3 (60%)	5 (100%)	1 (20%)
Total	161	42 (26.1%)	126 (78.3%)	67 (41.6%)	145 (90%)	103 (64%)

**Table 3:** Descriptive statistics of seed yield (SY) for standard varieties and samples from two breeding nurseries evaluated under field conditions in southeastern Kazakhstan (average values for 2024–2025). CVT: competitive variety testing; CTN: control testing nursery.

Descriptive Parameters	SY, t/ha		
	CVT	CTN	Standard
Valid	86	70	5
Mean	4.1	4.3	4.8
Std. Deviation	0.6	0.7	0.2
Variance	0.37	0.46	0.05
Minimum	2.9	3.1	4.8
Maximum	6.0	6.5	5.3

**Table 4:** Comparison of DNA identification data with phytopathological evaluation under an infection background.

Gene Combinations	Selected Lines		
	With Resistance-Associated Alleles	With Resistance-Associated Alleles and Field Resistance	With Field Resistance
Yellow rust			
*Yr5*/*Yr17*/*Yr18*	St. Egemen-20, Zhetysu, 20948-1, 20933-1, 20232-14, 9910, 9912, 9914-1, and 9829	9829 and 9910	K-1716-24, D305, 19434-5, 20061-12, 20389, 20060-1, 22416, 9904, 9909, 9910, 9923, 9926, 9927, 9829, 9810, and 21589-12
*Yr17*/*Yr18*	St. Tyalimi-80, 21730-1, 22596-3, D68, 9909, 9911, 9914, 9815, 9807, and 21325-22-1-1	9909	
*Yr5*/*Yr17*	Steklovidnaya-24, D304, D305, SWW 1/904, Sultan 2-2, D46, 21589-12, and 9819	D305 and 21589-12	
*Yr5*/*Yr18*	St. Almaly, 20197-17, 21303-1-3-1, 22255-2, 22257-3, 22208-2, K-2041-13-1, 19251-2, 20176-1, 18792-4, 18792-14, 9905, 9908, 9925, 22414-1, 22414-2, 22414-3, 22414-4, 9914-2, K-2041-13, and 9822	-	
*Yr5*/*Yr10*	952	-	
Leaf rust			
*Lr9*/*Lr34*/*Lr37*	Zhetysu, 21730-1, and 9910	9910	D302, D47, D306, 9910, 9909, and 9908
*Lr9*/*Lr34*	21110-1-3, 18717-5, and 9904	-	
*Lr34*/*Lr37*	20948-1, 22596-3, D68, 20232-14, 9909, 9911, 9912, 9914, 9914-1, 21325-22-1-1, 9815, and 9807	9909	
Common bunt			
*Bt8*/*Bt9*//*Bt10*/*Bt11*/*Bt12*	22596-3, 19251-2, 19434-5, 20060-3, 20232-14, 20388-3, 20841-2, 20841-17, 20948-8, 18952-1, 18792-14, 19405-1, 19251-3, 19439-3, and 19488-1	19251-2, 20232-14, 20841-2, and 20841-17	K-1716-24, Sultan-2-2, D68, 20061-12, 20114-16, 20232-14, 20841-2, 20841-17, 22382, 22416, 22414-1, 22414-2, 22548-3, 21266-3, 10/1013, 20982-2, 20437-9, 19251-2, 19980-6, 18411-1-1, 9828, 9908, 9909, 9926, 9905, and 9910
*Bt8*/*Bt9*/*Bt10*/*Bt11*	Steklovidnaya-24, Talimi-80, Zhetysu, 20948-1, 21730-1, 20933-1, 19051-4, and K-1127-7	-	
*Bt9*/*Bt10*/*Bt11*/*Bt12*	D68, Lira, D580, D306, K-1716-23, 20061-12, 20961-8, 20948-2-3, 21110-1-3, 22285-3-2, 22285-2-3, 21325-2-3-3, 22326-2, 22414-4, and 9804-1	D68 and 20061-12	
*Bt8*/*Bt10*/*Bt11*/*Bt12* *Bt8*/*Bt9*/*Bt11*/*Bt12*	20032-3-1, 20060-1, 22315-1, 20389, 20389-2, and 18792-4	-	
*Bt9*/*Bt11*/*Bt12*	10/1013, 18717-5, 20521-1, 21141-5-5, 22082-5, 20437-9, 21303-1-3-1, 22255-2, 22208-2, K-1716-24, D302, D305, 20114-13, 20114-16, 19051-11, 22376, 22416, 22382, 9908, 9909, 9914, 9925, 9927, 9928, 9930, 21688-5-2-3, 22258-2-1, 22285-3-1, 22285-3-3, 21325-2-3-2, 21325-22-1-1, 21325-22-1-2, 21325-22-1-3, and 9828	10/1013, 20437-9, K-1716-24, 20114-16, 22416, 22382, 9908, and 9909	

## Data Availability

The original contributions presented in this study are included in this article and its Supplementary Materials. Further inquiries can be directed to the corresponding author.

## Supplementary Materials

The following supporting information can be downloaded at https://www.mdpi.com/article/10.3390/plants15081258/s1. Figure S1: Identification of *Yr5* gene alleles using the S19M93 marker. Figure S2: Identification of Yr5 gene alleles using the S23M41 marker. Figure S3: Identification of Yr10 gene alleles using the Yr10SCAR marker. Figure S4: Identification of Yr10 gene alleles using the Xpsp3000 marker. Figure S5: Identification of Yr15 gene alleles using the Xgwm413 marker. Figure S6: Identification of Yr15 gene alleles using the Xbarc8 marker. Figure S7: Identification of Lr9 gene alleles using the SCS5550 marker. Figure S8: Identification of Lr34/Yr18/Sr57 gene complex alleles using the csLV34 marker. Figure S9: Identification of Lr37/Yr17/Sr38 gene alleles using the Ventriup-F and LN-2-R primers. Figure S10: Identification of Bt11 gene alleles using the Xgwm114 marker. Figure S11: Identification of Bt9 gene alleles using the Xgwm114 marker. Figure S12: Identification of Bt12 gene alleles using the Xgwm264 marker. Figure S13: Identification of Bt12 gene alleles using the Xgwm374 marker. Table S1: List of winter wheat samples. Results of genotyping, immunological assessment, and yield. Table S2: The results of the Mann–Whitney U test comparing DS (%) between wheat groups with and without resistance-associated alleles identified by DNA markers. Table S3: Results of the Chi-square (χ^2^) test comparing infection type (IT) among groups of wheat samples with different combinations of rust and common bunt resistance alleles, as determined by DNA marker-based genotyping. Table S4: Meteorological data (air temperature and precipitation) at the KRIAPG experimental fields during 2023–2025. Table S5: Genome location, primer sequences, and PCR conditions of the molecular markers used to identify rust resistance genes. Table S6: Genome location, primer sequences, and PCR conditions of molecular markers used to identify common bunt resistance genes.

## Abbreviations

The following abbreviations are used in this manuscript:

CIMMYT	International Maize and Wheat Improvement Center
CTN	Control Testing Nursery
CVT	Competitive Variety Testing
DS	Disease Severity
IT	Infection Type
KRIAPG	Kazakh Research Institute of Agriculture and Plant Growing
SCAR	Sequence-Characterized Amplified Region
SSR	Simple Sequence Repeat
STS	Sequence Tagged Site
SY	Seed Yield

## References

[1] Shiferaw B., Smale M., Braun H.J., Duveiller E., Reynolds M., Muricho G. (2013). Crops that feed the world 10. Past successes and future challenges to the role played by wheat in global food security. Food Secur..

[2] Igrejas G., Branlard G., Igrejas G., Ikeda T., Guzman C. (2020). The importance of wheat. Wheat Quality for Improving Processing and Human Health.

[3] Bohra A., Choudhary M., Bennett D., Joshi R., Mir R.R., Varshney R.K. (2024). Drought-tolerant wheat for enhancing global food security. Funct. Iintegr. Genom..

[4] Sommer R., Glazirina M., Yuldashev T., Otarov A., Ibraeva M., Martynova L., De Pauw E. (2013). Impact of climate change on wheat productivity in Central Asia. Agric. Ecosyst. Environ..

[5] Amalova A., Yermekbayev K., Griffiths S., Winfield M.O., Morgounov A., Abugalieva S., Turuspekov Y. (2023). Population structure of modern winter wheat accessions from Central Asia. Plants.

[6] FAO FAOSTAT: Wheat and Products. https://www.fao.org/faostat/en/#data/FBS.

[7] Rozewicz M., Wyzińska M., Grabinski J. (2021). The most important fungal diseases of cereals—Problems and possible solutions. Agronomy.

[8] Savary S., Willocquet L., Pethybridge S.J., Esker P., Mc Roberts N., Nelson A. (2019). The global burden of pathogens and pests on major food crops. Nat. Ecol. Evol..

[9] Bhavani S., Singh R.P., Hodson D.P., Huerta-Espino J., Randhawa M.S., Reynolds M.P., Braun H.J. (2022). Wheat Rusts: Current Status, Prospects of Genetic Control and Integrated Approaches to Enhance Resistance Durability. Wheat Improvement.

[10] Figueroa M., Hammond-Kosack K.E., Solomon P.S. (2018). A review of wheat diseases-a field perspective. Mol. Plant Pathol..

[11] Rahmatov M., Otambekova M., Muminjanov H., Rouse M.N., Hovmøller M.S., Nazari K., Steffenson B.J., Johansson E. (2019). Characterization of stem, stripe and leaf rust resistance in Tajik bread wheat accessions. Euphytica.

[12] Olivera P.D., Szabo L.J., Kokhmetova A., Morgounov A., Luster D.G., Jin Y. (2022). *Puccinia graminis* f. sp. *tritici* Population Causing Recent Wheat Stem Rust Epidemics in Kazakhstan Is Highly Diverse and Includes Novel Virulence Pathotypes. Phytopathology.

[13] Rsaliyev A.S., Rsaliyev S.S. (2018). Principal approaches and achievements in studying race composition of wheat stem rust. Vavilov J. Genet. Breed..

[14] Rsaliyev S., Rsaliyev A., Urazaliev R., Dubekova S., Serikbaykyzy A. (2025). Population composition and virulence of *Puccinia striiformis* f. sp. *tritici* in Kazakhstan. Plant Prot. Sci..

[15] Sekerova T., Tileubayeva Z., Ydyrys A., Aitzhanova M., Bakirova K., Mutlu M., Admanova G. (2021). Assessing Kazakhstani wheat varieties by yield indicators and resistance to rust. Int. J. Biol. Chem..

[16] Maulenbay A., Rsaliyev A. (2025). Assessing stem rust tolerance in commercial wheat varieties: Insights from field trials in Kazakhstan. Plant Prot. Sci..

[17] Kokhmetova A., Rsaliyev S., Atishova M., Kumarbayeva M., Malysheva A., Keishilov Z., Zhanuzak D., Bolatbekova A. (2021). Evaluation of wheat germplasm for resistance to leaf rust (*Puccinia triticina*) and identification of the sources of *Lr* resistance genes using molecular markers. Plants.

[18] Kokhmetova A., Rsaliyev A., Malysheva A., Atishova M., Kumarbayeva M., Keishilov Z. (2021). Identification of stripe rust resistance genes in common wheat cultivars and breeding lines from Kazakhstan. Plants.

[19] McIntosh R.A. (2024). Catalogue of Gene Symbols for Wheat. https://wheat.pw.usda.gov/GG3/wgc.

[20] Mishra K.K., Gahtyari N.C., Kant L., Kashyap P.L., Gupta V., Gupta O.P., Sendhil R., Gopalareddy K., Jasrotia P., Singh G.P. (2022). Common bunt and smuts in wheat and barley genetics, breeding, and management: Current status and future prospects. New Horizons in Wheat and Barley Research: Global Trends, Breeding and Quality Enhancement.

[21] Lunzer M., Dumalasová V., Pfatrisch K., Buerstmayr H., Grausgruber H. (2023). Common Bunt in organic wheat: Unravelling infection characteristics relevant for resistance breeding. Front. Plant Sci..

[22] Gurjar M.S., Kumar T.P.J., Shakouka M.A., Saharan M.S., Rawat L., Aggarwal R. (2023). Draft genome sequencing of *Tilletia caries* inciting common bunt of wheat provides pathogenicity-related genes. Front. Microbiol..

[23] Madenova A., Sapakhova Z., Bakirov S., Galymbek K., Yernazarova G., Kokhmetova A., Keishilov Z. (2021). Screening of wheat genotypes for the presence of common bunt resistance genes. Saudi J. Biol. Sci..

[24] Matanguihan J.B., Murphy K.M., Jones S.S. (2011). Control of common bunt in organic wheat. Plant Dis..

[25] Goates B.J. (2012). Identification of new pathogenic races of common bunt and dwarf bunt fungi, and evaluation of known races using an expanded set of differential wheat lines. Plant Dis..

[26] Tekin M. (2023). Genetic variation in Turkish bread wheat (*Triticum aestivum* L.) varieties for resistance to common bunt. Agronomy.

[27] Al-Maaroof E.M., Ali R.M., Aziz H.A.M.T.M. (2016). Searching for resistance sources to wheat common bunt disease and efficiency of *Bt* genes against *Tilletia tritici* and *T. laevis* populations. Agric. For..

[28] Veisz O., Szunics L., Szunics L. (2000). Effect of common bunt on the frost resistance and winter hardiness of wheat (*Triticum aestivum* L.) lines containing *Bt* genes. Euphytica.

[29] Aydoğdu M., Kaya Y. (2020). Reactions of spring wheat varieties to common bunt (*Tilletia laevis*) in Turkey. Cereal Res. Commun..

[30] Shahin A.A., Omara R.I., Omar H.A., El-Din H.S., Sehsah M.D., Essa T., Zayton M.A., Omar H.S. (2024). Evaluation of effectiveness resistance genes in wheat genotypes using marker-assisted selection for stripe rust resistance breeding. BMC Plant Biol..

[31] Jiyenbayeva K., Yessimbekova M., Bastaubayeva S., Morgounov A., Mukin K. (2025). Screening of winter wheat accessions from international variety trials for drought resistance in Southeastern Kazakhstan. Crops.

[32] Abugalieva A.I., Savin T.V., Kozhahmetov K.K., Morgounov A.I. (2021). Registration of wheat germplasm originating from wide crosses with superior agronomic performance and disease resistance. J. Plant Regist..

[33] Smith P.H., Hadifield J., Hart N.J., Koebner R.M.D., Boyd L.A. (2007). STS markers for the wheat yellow rust resistance gene *Yr5* suggest a NBS-LRR-type resistance gene cluster. Genome.

[34] Shao Y., Niu Y., Zhu L., Zhai W., Xu S., Wu L. (2001). Identification of an AFLP marker linked to the stripe rust resistance gene*Yr10* in wheat. Chin. Sci. Bull..

[35] Wang L.F., Ma J.X., Zhou R.H., Wang X.M., Jia J.Z. (2002). Molecular tagging of the yellow rust resistance gene *Yr10* in common wheat, P.I.178383 (*Triticm aestivum* L.). Euphytica.

[36] Peng J.H., Fahima T., Roeder M.S., Huang Q.Y., Dahan A., Li Y.C., Grama A., Nevo E. (2000). Highdensity molecular map of chromosome region harboring stripe-rust resistance genes *YrH52* and *Yr15* derived from wild emmer wheat, *Triticum dicoccoides*. Genetica.

[37] Murphy L.R., Santra D., Kidwell K., Yan G.P., Chen X.M., Campbell K.G. (2009). Linkage maps of wheat stripe rust resistance genes *Yr5* and *Yr15* for use in marker-assisted selection. Crop Sci..

[38] Gupta S.K., Charpe A., Koul S., Prabhu K.V., Haq Q.M. (2005). Development and Validation of Molecular Markers Linked to an *Aegilops umbellulata*-derived leaf rust resistance gene, *Lr9*, for marker-assisted selection in bread wheat. Genome.

[39] Lagudah E.S., McFadden H., Singh R.P., Huerta-Espino J., Bariana H.S., Spielmeyer W. (2006). Molecular genetic characterization of the *Lr34*/*Yr18* slow rusting resistance gene region in wheat. Theor. Appl. Genet..

[40] Helguera M., Khan I.A., Kolmer J., Lijavetzky D., Zhong-qi L., Dubcovsky J. (2003). PCR Assays for the *Lr37-Yr17-Sr38* cluster of rust resistance genes and their useto develop isogenic hard red spring wheat lines. Crop Sci..

[41] Cichy K., Goates B. Evaluation of molecular markers for common bunt resistance genes in diverse wheat genotypes. Proceedings of the ASA-CSSA-SSSA Annual Meeting, Pittsburg, PA, USA, 1–5 November 2009.

[42] Cota L.C., Pamfil D., Botez C., Grigoras M.A. (2010). Preliminary studies on microsatellite marker analysis of resistance to common bunt in several wheat genotypes (*Triticum aestivum* L.). Not. Bot. Horti Agrobot. Cluj-Napoca.

[43] Steffan P.M., Torp A.M., Borgen A., Backes G., Rasmussen S.K. (2017). Mapping of common bunt resistance gene *Bt9* in wheat. Theor. Appl. Genet..

[44] Laroche A., Demeke T., Gaudet D.A., Puchalski B., Frick M., McKenzie R. (2000). Development of a PCR marker for rapid identification of the *Bt-10* gene for common bunt resistance in wheat. Genome.

[45] Wang S., Knox R.E., DePauw R.M., Clarke F.R., Clarke J.M., Thomas J.B. (2009). Markers to a common bunt resistance gene derived from ‘Blizzard’ wheat (*Triticum aestivum* L.) and mapped to chromosome arm 1BS. Theor. Appl. Genet..

[46] Pratap A., Douglas C.A., Prajapati U., Kumari G., War A.R., Tomar R., Pandey A.K., Dubey S., Nair R., Schafleitner R., Lee S.H. (2020). Breeding progress and future challenges: Biotic stresses. The Mungbean Genome. Compendium of Plant Genomes.

[47] Robles-Zazueta C.A., Crespo-Herrera L.A., Pinera-Chavez F.J., Rivera-Amado C., Aradottir G.I. (2024). Climate change impacts on crop breeding: Targeting interacting biotic and abiotic stresses for wheat improvement. Plant Genome.

[48] Singh J., Chhabra B., Raza A., Yang S.H., Sandhu K.S. (2023). Important wheat diseases in the US and their management in the 21st century. Front. Plant Sci..

[49] Maulenbay A., Rsaliyev A. (2024). Fungal disease tolerance with a focus on wheat: A review. J. Fungi.

[50] Hovmøller M.S., Henriksen K.E. (2008). Application of pathogen surveys, disease nurseries and varietal resistance characteristics in an IPM approach for the control of wheat yellow rust. Eur. J. Plant Pathol..

[51] Osman M., He X., Singh R.P., Duveiller E., Lillemo M., Pereyra S.A., Westerdijk-Hoks I., Kurushima M., Yau S.-K., Benedettelli S. (2015). Phenotypic and genotypic characterization of CIMMYT’s 15th international Fusarium head blight screening nursery of wheat. Euphytica.

[52] Leharwan M., Singh A.K., Kumar A., Kashyap P.L., Kumar S., Singh R., Gangwar O.P. (2025). Phenotyping and deciphering genetic resistance to yellow rust in wheat through marker-assisted analysis. Physiol. Mol. Plant Pathol..

[53] Gultyaeva E., Shaydayuk E. (2023). Resistance of modern Russian winter wheat cultivars to yellow rust. Plants.

[54] Draz I.S., Abou-Elseoud M.S., Kamara A.E.M., Alaa-Eldein O.A.E., El-Bebany A.F. (2015). Screening of wheat genotypes for leaf rust resistance along with grain yield. Ann. Agric. Sci..

[55] Zewdu D., Solomon T., Alemu G., Dabi A., Geleta N., Sime B., Duga R., Delesa A., Zegeye H., Getamesay A. (2024). Phenotypic Screening of Bread Wheat (*Triticum* spp L.) Germplasm Collection for Yellow and Leaf Rust Disease Resistance. Int. J. Econ. Plants.

[56] Song L., Wang R., Yang X., Zhang A., Liu D. (2023). Molecular markers and their applications in marker-assisted selection (MAS) in bread wheat (*Triticum aestivum* L.). Agriculture.

[57] Collard B.C., Mackill D.J. (2008). Marker-assisted selection: An approach for precision plant breeding in the twenty-first century. Philos. Trans. R. Soc. B Biol. Sci..

[58] Lai H., Shen Y., Yang H., Fernando D.W.G., Ren C., Deng F., Lu Y., Sun N., Chen L., Li G. (2024). Comparative analysis of stripe rust resistance in seedling stage and Yr gene incidence in spring and winter wheat from Xinjiang, China. Front. Plant Sci..

[59] Muleta K.T., Rouse M.N., Rynearson S., Chen X., Buta B.G., Pumphrey M.O. (2017). Characterization of molecular diversity and genome-wide mapping of loci associated with resistance to stripe rust and stem rust in Ethiopian bread wheat accessions. BMC Plant Biol..

[60] Malysheva A., Kokhmetova A., Urazaliev R., Kumarbayeva M., Keishilov Z., Nurzhuma M., Bolatbekova A., Kokhmetova A. (2023). Phenotyping and identification of molecular markers associated with leaf rust resistance in the wheat germplasm from kazakhstan, CIMMYT and ICARDA. Plants.

[61] Kokhmetova A., Madenova A., Kampitova G., Urazaliev R., Yessimbekova M., Morgounov A., Purnhauser L. (2016). Identification of Leaf Rust Resistance Genes in Wheat Cultivars Produced in Kazakhstan. Cereal Res. Commun..

[62] Yerzhebayeva R.S., Bazylova T.A., Babissekova D.I., Amangeldiyeva A.A., Tajibayev D.G., Ydyrys A. (2020). studying a spring triticale collection for resistance to leaf and stem rusts using allele-specific markers. Cytol. Genet..

[63] Liatukas Ž., Ruzgas V. (2008). Resistance genes and sources for the control of wheat common bunt (*Tilletia tritici* (DC.) Tul.). Biologija.

[64] Bryant R.R.M., McGrann G.R.D., Mitchell A.R., Schoonbeek H.-J., Boyd L.A., Uauy C., Dorling S., Ridout C.J. (2014). A change in temperature modulates defence to yellow (stripe) rust in wheat line UC1041 independently of resistance gene *Yr36*. BMC Plant Biol..

[65] (2011). Methodology for Variety Trial of Agricultural Plants.

[66] Wilcoxson R.D., Saari E.E., Hettel G., McNab A., Mexico D.F. (1996). Bunt and Smut Diseases of Wheat: Concepts and Methods of Disease Management.

[67] Mourad A.M., Morgounov A., Baenziger P.S., Esmail S.M. (2022). Genetic variation in common bunt resistance in synthetic hexaploid wheat. Plants.

[68] Roelfs A.P., Singh R.P., Saari E.E., Hettel G. (1992). Rust Diseases of Wheat: Concepts and Methods of Disease Management.

[69] (1986). Rust Scoring Guide (Handbook).

[70] Tehseen M.M., Tonk F.A., Tosun M., Randhawa H.S., Kurtulus E., Ozseven I., Akin B., Nur Zulfuagaoglu O., Nazari K. (2022). QTL mapping of adult plant resistance to stripe rust in a doubled haploid wheat population. Front. Genet..

[71] Peterson R.F., Campbell A.B., Hannah A.E. (1948). A diagrammatic scale for estimating rust intensity of leaves and stem of cereals. Can. J. Res. Sect..

[72] Beck H.E., McVicar T.R., Vergopolan N., Berg A., Lutsko N.J., Dufour A., Zeng Z., Jiang X., van Dijk A.I.J.M., Miralles D.G. (2023). High- resolution (1 km) Koppen-Geiger maps for 1901–2099 based on constrained CMIP6 projections. Sci. Data.

[73] Amangaliev B.M., Zhusupbekov E.K., Malimbaeva A.Z., Batyrbek M., Rustemova K.U., Tabynbayeva L.K. (2023). Dynamics of fertility indicators of light-chestnut soil and oil flax productivity under bogarian conditions of Southeast Kazakhstan. SABRAO J. Breed. Genet..

[74] Delaporta S.L., Wood J., Hicks J.B. (1983). A plant DNA minipreparation. Version II. Plant Mol. Biol. Rep..

[75] JASP Version 0.95.4. https://jasp-stats.org/.

