# Supplementary material for: Integrating Phenotypic and Genotypic Approaches to Select Rust- and Common Bunt-Resistant Advanced Winter Wheat Breeding Lines
Source: Plants (Basel). 2026 Apr 19;15(8):1258. doi: 10.3390/plants15081258 (PMC13120159; doi:10.3390/plants15081258)
Supplement: Supplementary file 1 [file plants-15-01258-s001.zip › Supplementary Figures S1- S13, Tables S2-S6.pdf]

Supplementary Materials to the paper

Gaziza Zhumaliyeva, Bakyt Ainebekova, Tamara Bazylova, Assel Jenisbayeva, Ayazhan Kosshybay, Saltanat Dubekova, Raushan Yerzhebayeva. Integrating Phenotypic and Genotypic Approaches for Select-ing Rust and Common Bunt Resistant Advanced Winter Wheat Breeding Lines. Plants 2026.

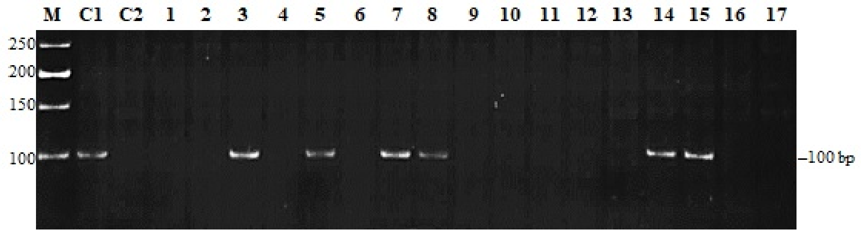

Figure S1. Identification of *Yr5* gene alleles in the winter wheat breeding lines using S19M93 marker: M, Molecular weight marker (Step50 Plus); C1, positive control IL №7-20ENTRY, C2, negative control Avocet'S', 1, 21144-4-1; 2, 22180-1; 3, 21692-2-1; 4, 22161-1; 5, 22208-2; 6, K-1676; 7, K-1127-7; 8, 2041-13; 9, 2041-7; 10, 1716-23; 11, 22315-1; 12, K-1716-24; 13, D302; 14, D304; 15, D305; 16, D306; 17, D307.

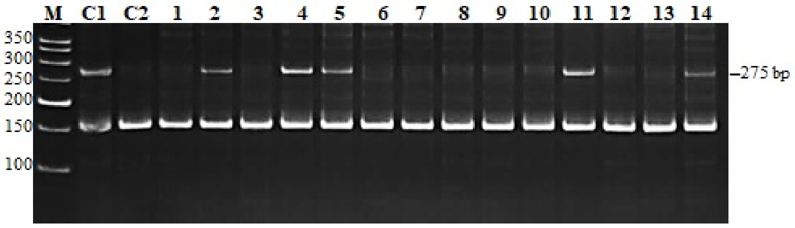

Figure S2. Identification of *Yr5* gene alleles in the winter wheat breeding lines using S23M41 marker: M, Molecular weight marker (Step50 Plus); C1, positive control IL №7-20ENTRY, C2, negative control Avocet'S', 1, 20948-8; 2, 18792-4; 3, 18952-1; 4, 18792-14; 5, 19401-7; 6, 19405-1; 7, 19051-11; 8, 19251-3; 9, 19439-3; 10, 19488-1; 11, 19995-2; 12, 20961-8; 13, 18411-1-1; 14, 20032-3.

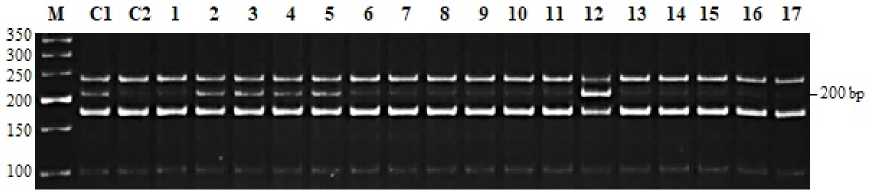

Figure S3. Identification of *Yr10* gene alleles in the winter wheat breeding lines using Yr10SCAR marker: M, Molecular weight marker (Step50 Plus); C1, positive control IL №12-20ENTRY, C2, negative control Avocet'S', 1, 21144-4-1; 2, 22180-1; 3, 21692-2-1; 4, 22161-1; 5, 22208-2; 6, K-1676; 7, K-1127-7; 8, K-2041-13-1; 9, 19670-1; 10, 1716-24; 11, 20060-3; 12, 1716-24; 13, D302; 14, D304; 15, D305; 16, D306; 17, D307.

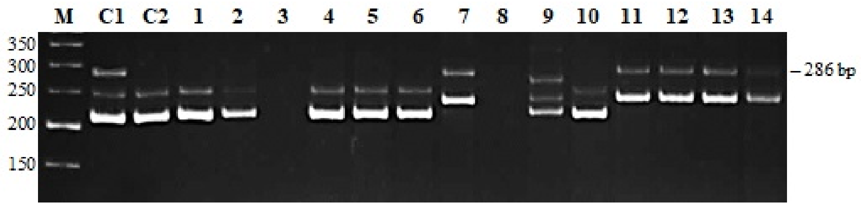

Figure S4. Identification of *Yr10* gene alleles in the winter wheat breeding lines using Xpsp3000 marker: M, Molecular weight marker (Step50 Plus); C1, positive control IL №12-20ENTRY, C2,

negative control Avocet'S', 1, 19051-4; 2, 21144-4-1; 3, 22180-1; 4, 21692-2-1; 5, 22161-1; 6, 22208-2; 7, K-1676; 8, K-1127-7; 9, K-2041-13-1; 10, 22315-1; 11, K-1716-24; 12, 20437-9; 13, 952; 14, D302.

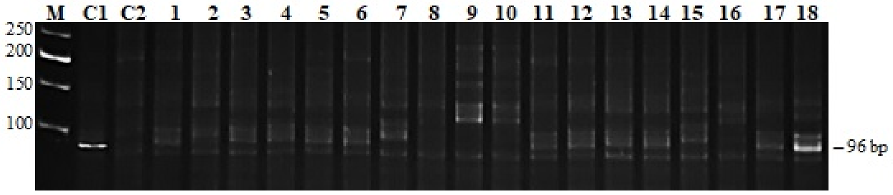

Figure S5. Identification of *Yr15* gene alleles in the winter wheat breeding lines using Xgwm413 marker: M, Molecular weight marker (Step50 Plus); C1, positive control IL №13-20ENTRY, C2, negative control Almaly, 1, 19488-22; 2, 19670-1; 3, 20841-2; 4, 20841-17; 5, 20060-3; 6, 20061-12; 7, 20114-13; 8, 20114-16; 9, 20009-6; 10, 20156-3; 11, 20156-4; 12, 20176-1; 13, 20232-14; 14, 20388-3; 15, 20388-7; 16, 19980-6; 17, 20389; 18, D68.

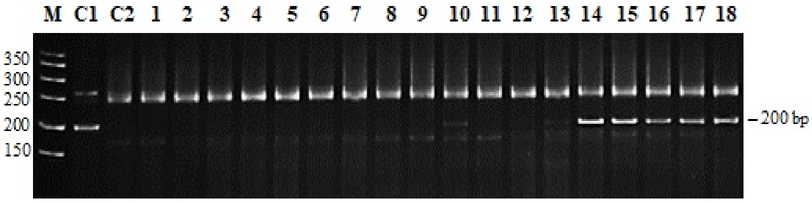

Figure S6. Identification of *Yr15* gene alleles in the winter wheat breeding lines using Xbarc8 marker: M, Molecular weight marker (Step50 Plus); C1, positive control IL №13-20ENTRY, C2, negative control Almaly, 1, 22414-4; 2, 22547; 3, 22548-1; 4, 22548-3; 5, 9914-1; 6, 9914-2; 7, K-2041-13; 8, 9829; 9, 9828; 10, 9827; 11, 9825; 12, 9822; 13, 9821; 14, 9910; 15, 9911; 16, 9927; 17, 9928; 18, K-1716-23.

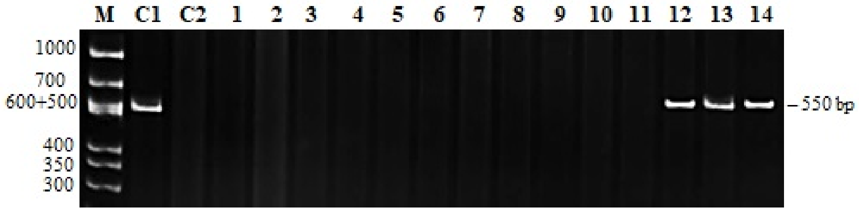

Figure S7. Identification of *Lr9* gene in the winter wheat breeding lines using $SCS5_{550}$ marker: M, Molecular weight marker (Step50 Plus); C1, positive control cv. Phyton *Lr9*, and C2, negative control Almaly, 1, St. Steklovidnaya-24; 2, 19488-22; 3, 19670-1; 4, 20060-3; 520061-12; 6, 20060-1; 7, 20114-13; 8, 20114-13; 9, 20009-6; 10, 20156-3; 11, 20156-4; 12, 18717-5; 13, 21730-1; 14, 21110-1-3;

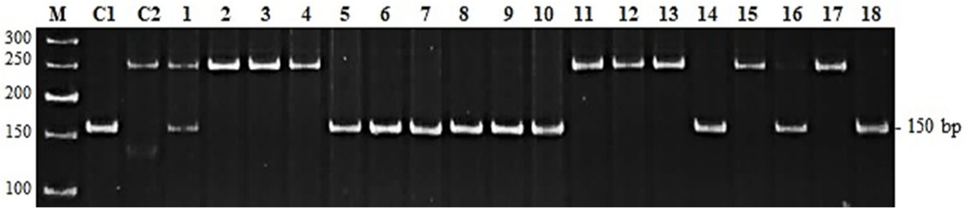

Figure S8. Identification of *Lr34*/*Yr18*/*Sr57*/*Pm38* gene complex in the winter wheat breeding lines using csLV34 marker: M, Molecular weight marker (Step50 Plus); C1, positive control cv. NIL-Thatcher-Lr34-PI58548 (RL6058), and C2, negative control Morocco, 1, St. Almaly; 2, St. Egemen-20; 3, 20521-1; 4, 18411-1; 5, 18410-1; 6, 21266-3; 7, 20197-17; 8, 20052-9-4; 9, 21141-5-5; 10, 20982-2; 11, 21203-11-3; 12, 21583-4; 13, 22030-10; 14, 22082-5; 15, D580; 16, 20437-9; 17, 952; 18, 21106-2-5.

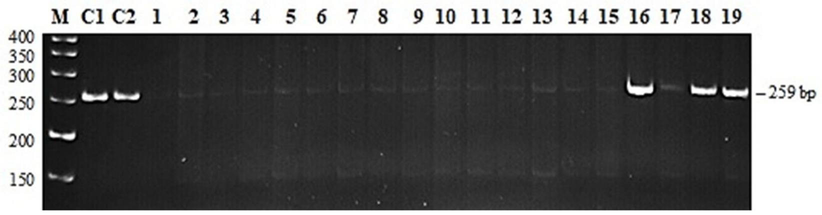

Figure S9. Identification of *Lr37*/*Sr38*/*Yr17* gene in the winter wheat breeding lines using Ventriup-F and LN-2-R primers: M, Molecular weight marker (Step50 Plus); C1, positive control cv. NIL-THATCHER-Lr37-VPM (RL6081), C2, positive control cv. Trident Sr38, 1, 22257-3; 2, 22315-1; 3, 19051-4; 4, 21144-4-1; 5, 22180-1; 6, 21692-2-1; 7, 22161-1; 8, 22208-2; 9, K-1676; 10, K-1127-7; 11, K-2041-13-1; 12, K-1716-24; 13, 19251-2; 14, 19434-5; 15, 19488-22; 16, D302; 17, D304; 18, D305; 19, D306.

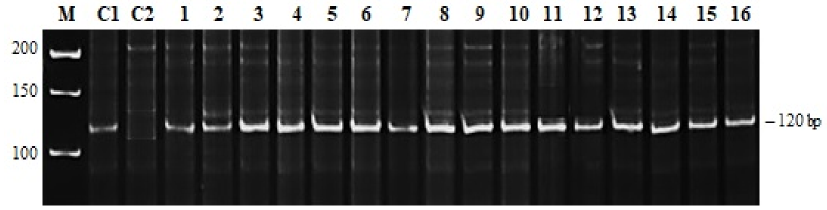

Figure S10. Identification of *Bt11* gene in the winter wheat breeding lines using Xgwm114 marker: M, Molecular weight marker (Step50 Plus); C1, positive control IL M82-2123, C2, negative control Bogarnaya 56, 1, 19488-22; 2, 19670-1; 3, 20841-2; 4, 20841-17; 5, 20060-3; 6, 20061-12; 7, 20114-13; 8, 20114-16; 9, 20009-6; 10, 20156-3; 11, 20841-17; 12, 20176-1; 13, 20232-14; 14, 20388-3; 15, 20388-7; 16, 19980-6; 17, 20389; 18, 20389-2.

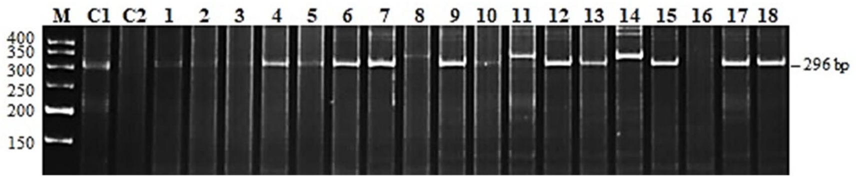

Figure S11. Identification of *Bt9* gene in the winter wheat breeding lines using Xgwm114 marker: M, Molecular weight marker (Step50 Plus); C1, positive control IL M77-1140, C2, negative control Bogarnaya 56, 1, 22372Kl; 2, 19995-2; 3, 18411-1-1; 4, 19488-1; 5, 19439-3; 6, 19251-3; 7, 19051-11; 8, 20388-7; 9, 19405-1; 10, 19401-7; 11, 9902; 12, 9904; 13, 9904; 14, 9908; 15, 9905; 16,9911; 17, 9910; 18, 9914.

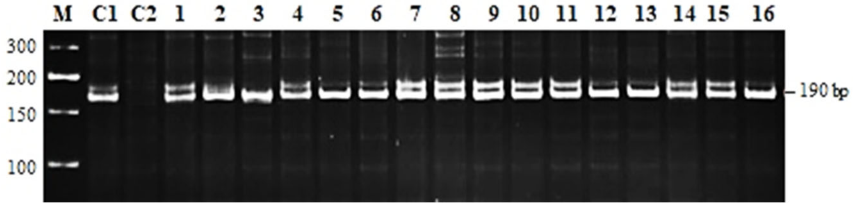

Figure S12. Identification of *Bt12* gene in the winter wheat breeding lines using Xgwm264 marker: M, Molecular weight marker (Step50 Plus); C1, positive control IL M77-1140, C2, negative control Bogarnaya 56, 1, 19488-22; 2, 19434-5; 3, 20841-2; 4, 20841-17; 5, 20060-3; 6, 20061-12; 7, 20114-13; 8, 20114-16; 9, 20009-6; 10, 20156-3; 11, 20841-17; 12, 20176-1; 13, 20232-14; 14, 20388-3; 15, 20388-7; 16, 19980-6.

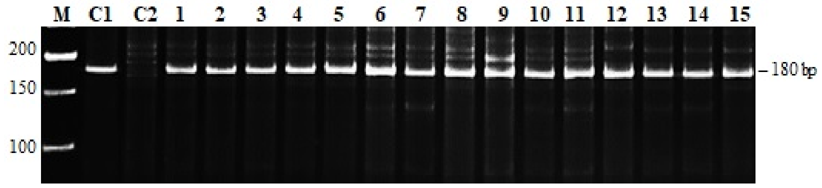

Figure S13. Identification of *Bt12* gene in the winter wheat breeding lines using Xgwm374 marker: M, Molecular weight marker (Step50 Plus); C1, positive control IL M77-1140, C2, negative control Bogarnaya 56, 1, 20841-17; 2, 20948-8; 3, 18792-4; 4, 18952-1; 5, 18792-14; 6, 19401-7; 7, 19405-1; 8, 19051-11; 9, 19251-3; 10, 19439-3; 11, 19488-1; 12, 19995-2; 13, 20961-8; 14, 18411-1; 15, 20032-3.

Table S2. The results of the Mann–Whitney U test comparing DS (%) between wheat groups with and without resistance-assosiated alleles identified by DNA markers

Gene	Molecular Markers	U	p	Rank-Biserial Correlation	SE Rank-Biserial Correlation
		**Yellow rust resistance genes**			
Yr5	S19M93-100	3106	0.55	-0.05	0.095
	S23M41-275	3003	0.65	-0.04	0.095
Yr10	Cs-200	2831	0.11	-0.16	0.104
	Xpsp3000	599.5	0.59	0.11	0.196
Yr15	Xbarc8	816.5	0.23	0.19	0.161
	Xgwm413	594.5	0.03	-0.55	0.257
Yr18	csLV34	3397	0.33	-0.09	0.092
Yr17	Ventriup-F, LN-2-R	1718	0.01	0.26	0.106
		**Leaf rust resistance genes**			
Lr9	$SCS5_{550}$	80.0	0.02	0.67	0.286
Lr37	Ventriup-F, LN-2-R	810.5	0.02	0.32	0.133
Lr34	csLV34	1729	0.20	0.13	0.103
		**Common bunt resistance genes**			
Bt8	Xgwm114	2526	0.49	-0.07	0.105
Bt9	Xgpw7433	1665	0.05	0.19	0.112
Bt10	FSD/RSA	2955	0.99	0.001	0.094
Bt11	Xgwm114	880.5	0.16	0.21	0.152
Bt12	Xgwm374	1442	0.02	-0.36	0.156
	Xgwm264	2464	0.79	0.03	0.101

Table S3. Results of the chi-square (χ^2) test comparing infection type (IT) among groups of wheat samples with different combinations of rust and common bunt resistance alleles, as determined by DNA marker-based genotyping.

Group	X^2	df	p
Groups with *Bt* genes	108.7	120	0.762
Groups with *Yr* genes	32.3	32	0.453
Groups with *Lr* genes	23.7	30	0.322

Table S4. Meteorological data (air temperature and precipitation) at the KRIAPG experimental fields during 2023–2025.

Month	Year	Air temperature, °C		Precipitation, mm.	
		monthly	multi-year average	monthly	multi-year average
March	2023	8.4	4.4	61.2	65.6
April		11.9	11.5	68.2	108.5
May		17.2	16.8	43.4	97.1
June		24.6	21.4	4.3	58.4
July		27.1	23.9	33.6	56.9
March	2024	5.4	-4.4	135.5	65.6
April		12.8	10.9	111.3	110.6
May		17.6	16.3	121.2	98.4
June		24.5	21.2	19.7	59.9
July		25.0	23.7	85.2	56.9
March	2025	6.2	2.8	76.8	64.7
April		15.7	10.9	57.2	110.6
May		20.8	16.3	80.4	98.0
June		25.6	21.2	17.1	59.9
July		27.7	23.7	9.6	56.9

Table S5. Genome location, primers sequences, and PCR conditions of molecular markers used to identify rust resistance genes

Gene	Chromosome	Source of gene	Molecular marker			Product size (bp)	References
			Primer	Sequence of Primer (5′→3′)	PCR Amplification Conditions		
1	2	3	4	5	6	7	
Yr5	2BL	*Triticum spelta L. var. album Perc.*	S19M93-100	AATTGGGACCGAGAGACG<br>TCTTGCAGCTCCAAAACCT	94°C-3 min, 30 cycles (94°C-20 s, 58°C-20 s, 72°C-45 s), 72°C-1 min.	100	[33]
			S23M41-275	TCAACGGAACCTCCAATTTC<br>AGGTAGGTGTTCCAGCTTGC		275	[33]
Yr10	1BS	*Turkish wheat landrace PI 178383*	Scar-200	CTGCAGAGTGACATCATACA<br>TCGAACTAGTAGATGCTGGC	94°C-3 min, 30 cycles (94°C-10 s, 57°C-10 s, 72°C-15 s), 72°C-5 min.	200+/180 -	[34]
			Xpsp3000	GCAGACCTGTGTCATTGGTC<br>GATATAGTGGCAGCAGGATACG	94°C-3 min, 30 cycles (94°C-10 s, 55°C-10 s, 72°C-15 s), 72°C-5 min.	286+/240-	[34, 35]
Yr15	1BS	*Triticum dicoccoides*	Xbarc8	GCGGGAATCATGCATAGGAAAACAGAA<br>GCGGGGGCGAAACATACACATAAAAACA	94°C-3 min, 30 cycles (94°C-20 s, 52°C-20 s, 72°C-45 s), 72°C-1 min.	200	[36, 37]
			Xgwm413	TTTTTGGCTTATTAGACTGACTT<br>TTGCCATAAAATACAAAATCC′	94°C-3 min, 30 cycles (94°C-15 s, 60°C-20 s, 72°C-25 s), 72°C-5 min.	96+/100-	[36, 37]
Lr9	6BL	*Aegilops umbellulata*	SCS5	TGCGCCCTTCAA AGGAAG<br>TGCGCCCTTCTG AACTGTAT	94°C-5 min, 30 cycles (94°C-30 s, 64°C-30 s, 72°C-1 min), 72°C-7 min.	550	[38]
Lr34/Yr18/Sr57	7DS	*Triticum aestivum*	csLV34	GTTGGTTAAGACTGGTGATGG<br>TGCTTGCTATTGCTGAATAGT	94°C-5 min, 30 cycles (94°C-45 s, 60°C-30 s, 72°C-60 s), 72°C-7 min.	150+/229 -	[39]
Lr37/SR38/Yr17	2A	*Aegilops ventricosa*	Ventriup-F<br>LN-2-R	AGGGCTACTGACCAAGGCT<br>TGCAGCTACAGCAGTAGTATGTACACAAAA	94°C-3 min, 30 cycles (94°C-30 s, 65°C-35 s, 72°C-40 s), 72°C-5 min.	259	[40]

Table S6. Genome location, primers sequences and PCR conditions of molecular markers used to identify common bunt resistance genes

Gene	Marker	Chromosome	Molecular marker			Product size (bp)	References
			Primer	Sequence of Primer (5′→3′)	PCR Amplification Conditions		
1	2	3	4	5	6	7	
Bt8<br>*Bt11*	SSR	6DS	Xgwm114	ACAAACAGAAAATCAAAACCCG<br>ATCCATCGCCATTGGAGTG	95°C – 3 min, 30 cycles (94°C – 30 s, 58°C – 30 s, 72°C – 30 s), 72°C – 5 min.	180<br>120	[41]
Bt9	SSR	6DL	Xgpw7433	GTACATGGAAAGAGACCAACACCA<br>CGCTGAGCAAGGACGATAG	95°C – 3 min, 30 cycles (94°C – 30 s, 59°C – 30 s, 72°C – 30 s), 72°C – 5 min.	296	[43]
Bt10	SCAR	6DS	FSD/RSA	GTTTTATCTTTTTATTTC<br>CTCCTCCCCCCA	95°C – 3 min, 30 cycles (94°C – 30 s, 37°C – 30 s, 72°C – 30 s), 72°C – 5 min.	275/300	[44]
Bt12	SSR	7DS	Xgwm264	GAGAAACATGCCGAACAACA<br>GCATGCATGAGAATAGGAACTG	95°C – 3 min, 30 cycles (94°C – 40 s, 60°C – 60 s, 72°C – 40 s), 72°C – 1 min 15 s.	190	[45]
			Xgwm374	ATAGTGTGTTGCATGCTGTGTG<br>TCTAATTAGCGTTGGCTGCC		180	[45]
